# Melittin as a promising anti-protozoan peptide: current knowledge and future prospects

**DOI:** 10.1186/s13568-021-01229-1

**Published:** 2021-05-13

**Authors:** Hamed Memariani, Mojtaba Memariani

**Affiliations:** 1 Skin Research Center, Shahid Beheshti University of Medical Sciences, Tehran, Iran; 2 Biotechnology Research Center, Pasteur Institute of Iran, Tehran, Iran; 3 Department of Pathobiology, School of Public Health, Tehran University of Medical Sciences, Tehran, Iran

**Keywords:** Melittin, Anti-protozoan effects, *Leishmania*, *Plasmodium*, *Trypanosoma*

## Abstract

Protozoan diseases such as malaria, leishmaniasis, Chagas disease, and sleeping sickness still levy a heavy toll on human lives. Deplorably, only few classes of anti-protozoan drugs have thus far been developed. The problem is further compounded by their intrinsic toxicity, emergence of drug resistance, and the lack of licensed vaccines. Thus, there is a genuine exigency to develop novel anti-protozoan medications. Over the past years, melittin, the major constituent in the venom of European honeybee *Apis mellifera*, has gathered the attention of researchers due to its potential therapeutic applications. Insofar as we are aware, there has been no review pertinent to anti-protozoan properties of melittin. The present review outlines the current knowledge about anti-protozoan effects of melittin and its underlying mechanisms. The peptide has proven to be efficacious in killing different protozoan parasites such as *Leishmania*, *Plasmodium*, *Toxoplasma*, and* Trypanosoma* in vitro*.* Apart from direct membrane-disruptive activity, melittin is capable of destabilizing calcium homeostasis, reducing mitochondrial membrane potential, disorganizing kinetoplast DNA, instigating apoptotic cell death, and induction of autophagy in protozoan pathogens. Emerging evidence suggests that melittin is a promising candidate for future vaccine adjuvants. Transmission-blocking activity of melittin against vector-borne pathogens underscores its potential utility for both transgenic and paratransgenic manipulations. Nevertheless, future research should focus upon investigating anti-microbial activities of melittin, alone or in combination with the current anti-protozoan medications, against a far broader spectrum of protozoan parasites as well as pre-clinical testing of the peptide in animal models.

## Keypoints

- Melittin targets cell membrane and intracellular components of protozoan pathogens.
- Due to adjuvant properties of melittin, it has the potentiality for developing anti-leishmania vaccine.
- Transgenic mosquitoes expressing melittin may offer opportunities for controlling malaria.

## Introduction

Protozoan infections imperil the lives of almost one-third of the world’s population. Malaria, visceral leishmaniasis (kala-azar), Chagas disease (American trypanosomiasis), and sleeping sickness (African trypanosomiasis) still remain as dreadful scourges to mankind, particularly in tropical and sub-tropical regions (Sbaraglini et al. 2016; Norman et al. 2020). Protozoans are a diverse, polyphyletic group of heterotrophic unicellular eukaryotic organisms (Karpiyevich and Artavanis-Tsakonas 2020). In view of the fact that they share many metabolic pathways with their mammalian hosts, drug development against these pathogens has long been an excruciating task for pharmaceutical industry. For this reason, only a handful of new medications with anti-protozoal activities have come on the market over the course of past decades (Müller and Hemphill 2016). The problem is further exacerbated by their intrinsic toxicity, emergence of drug resistance, ineffective vector control interventions, and the lack of licensed vaccines (Capela et al. 2019). To truly address these encumbrances, innovative approaches and frugal solutions are required. Perhaps counter-intuitively, animal venoms could serve as an untapped source of novel anti-microbial drug candidates (Memariani and Memariani 2020).

Since antiquity, various honeybee products including honey, royal jelly, beeswax, propolis, bee pollen, and bee venom have been exploited for not only nutritional purposes, but also curative intentions (Cornara et al. 2017; Duffy et al. 2020). Bees are armed with stings and potent venoms to fend off intruders (Walker et al. 2020). The venom of European honeybee (*Apis mellifera*) is replete with a complex farrago of biologically active substances such as peptides, enzymes, and amines (Dotimas and Hider 1987). Honeybee venom has been extensively used as a traditional anti-inflammatory remedy for a myriad of illnesses such as dermatological maladies (Kim et al. 2019), multiple sclerosis (Hauser et al. 2001), rheumatoid arthritis (Lee et al. 2014), and chronic pains (Seo et al. 2017), though the precise mechanism of action at the cellular level has not been fully realized so far.

Prominent among the honeybee venom components is melittin, an amphipathic hexacosapeptide, which makes up around half of the dry weight (Maulet et al. 1982). Despite having a conspicuously hydrophobic amino acid composition, melittin represents a net charge of + 6 at physiological pH due to the existence of lysine and arginine residues (Dempsey 1990). In an aqueous solution of low concentration and low ionic strength, the peptide displays a random coil conformation (Vogel 1981). It assumes an α-helical structure in the presence of various detergent molecules or lipid membranes (Knöppel et al. 1979; Lauterwein et al. 1979). Melittin is thought to be monomeric at low peptide concentration, while it is associated as a tetrameric aggregate under certain conditions such as high salt concentration and/or high pH (Hall et al. 2011). Another feature of melittin is its ability to induce pore formation in natural and artificial lipid membranes (van den Bogaart et al. 2008). This membrane-disruptive activity of melittin may culminate in cell lysis (Memariani et al. 2020a). Further explanations concerning all features of melittin are beyond the scope of this review. Therefore, we refer interested readers to other thorough publications (Raghuraman and Chattopadhyay 2007; Carpena et al. 2020; Hong et al. 2019).

As far back as the early 1950s, the existence of melittin in the honeybee venom became apparent when the direct hemolysin was electrophoretically separated from the indirect hemolysin phospholipase A (Neumann et al. 1952; Habermann 1972). The first fundamental investigation on anti-microbial properties of melittin was carried out by Fennell et al. (1967), who reported that a penicillin-resistant isolate of *Staphylococcus aureus* was susceptible to bee venom and its melittin fraction. It was not until the late 1980s that melittin was shown to be cytotoxic to trypanosomatid protozoan parasites (Azambuja et al. 1989). In particular, the past two decades have witnessed a good deal of interest on biological effects and modes of action of melittin against different protozoan parasites. The therapeutic potentiality of melittin has also been emphasized repeatedly in recent years.

Insofar as we are aware, no review has discussed the anti-protozoan activities of melittin and its underlying mechanisms. This compendious review is thus the first endeavor to synopsize the literature available on the subject. For this purpose, literature searches with PubMed and Google Scholar using words [melittin] and [protozoa] or [antiprotozoal activity] or [*Leishmania*] or [*Plasmodium*] or [*Toxoplasma*] or [*Trypanosoma*] for published English-language papers from inception until the end of 2020 were carried out by the authors. For the sake of readers' convenience, a brief description of relevant pathogens is given at the outset of each section.

## Anti-protozoan properties of melittin

### *Leishmania* spp.

Leishmaniasis is a sandfly-transmitted disease caused by obligate intracellular parasite of the genus *Leishmania* (Roatt et al. 2020). The dimorphic life-cycle of *Leishmania* relies upon continuous shuttling between an invertebrate vector and a mammalian host. Within the digestive tract of sandfly, the pathogen exists as extracellular flagellated promastigotes. Contrariwise, intracellular non-motile amastigotes survive and proliferate inside host’s phagocytes (Steverding 2017). Given that different species of *Leishmania* are morphologically indiscernible, a variety of techniques based on molecular methods, isoenzyme analysis, or monoclonal antibodies have so far been developed for the differentiation of the pathogenic species (Thakur et al. 2020). The clinical manifestations of leishmaniasis are not only dependent on the species of the pathogen, but also on the immunological status and genetically determined responses of patients. These range from self-resolving cutaneous ulcers to mutilating mucocutaneous lesions and even to severe, life-menacing visceral infections. In this respect, *L. major* and *L. tropica* are the main dermotropic species, whereas visceral leishmaniasis is predominantly caused by *L. donovani* or *L. infantum* (Roatt et al. 2020). It has been stated that roughly 1.5 to 2 million new cases of leishmaniasis occur annually, of whom 70,000 perished as a result of complications (Torres-Guerrero et al. 2017). Pentavalent anti-monials are still considered as the first line of treatment for various forms of leishmaniasis, though other new medications including amphotericin B (in deoxycholate or liposomal formulations), miltefosine, and paromomycin are now available (Roatt et al. 2020). These therapies have certain detrimental effects or pharmacological liabilities that may cause treatment failure or relapse of the disease (Roatt et al. 2020).

The half lethal dose (LD_50_) of melittin towards *L. donovani* (2 × 10^7^ promastigote/mL) has been estimated to be 0.3 μM, as determined by 3-(4,5-dimethylthiazol-2-yl)-2,5-diphenyltetrazolium bromide (MTT) assay (Díaz-Achirica et al. 1998). Intriguingly, a lower concentration of melittin (0.2 μM) was found to be sufficient to raise intracellular concentration of free calcium ([Ca^2+^]i) from 100 to around 440 nM when the *L. donovani* promastigotes were suspended in medium comprising 2 mM CaCl_2_ (Catisti et al. 2000). In addition, a dose-dependent enhancement of [Ca^2+^]i in promastigotes was reported at melittin concentrations ranging from 0.1 to 0.5 μM. Other species of *Leishmania* have also been surveyed for their vulnerability to melittin treatment (Table 1). According to a study conducted by Pereira et al. (2016), the concentration of melittin yielding half-maximal inhibition (IC_50_) of promastigotes and intracellular amastigotes of *L. infantum* were 28.29 and 1.40 μg/mL, respectively. In the case of *L. major* (2 × 10^6^ promastigotes/mL), the median effective concentration (EC_50_) of melittin, representing a peptide concentration for inducing cell death in 50% of the treated parasites, was found to be 74.01 ± 1.27 μg/mL. Nevertheless, melittin concentrations up to 100 μg/mL were not adequate to reach the EC_50_ against *L. panamensis* promastigotes (Pérez-Cordero et al. 2011). These results point out that *L. panamensis* promastigotes are more resistance to melittin exposure as compared with *L. major* promastigotes in vitro. One study also revealed that the concentration required to diminish the infection rate of internalized *L. panamensis* amastigotes by 50% (EC_50_i) was greater or equal to 10 μg/mL (Pérez-Cordero et al. 2011).

**Table 1:** Anti-microbial effects of melittin against various protozoan parasites. Developmental forms of tested protozoa, methodologies, and key findings of the relevant studies have also been included

Protozoan parasites	Identifier	Developmental forms	Methods	Key findings	References
*Leishmania donovani*	S-2 strain	Promastigotes	Evaluation of Ca^2+^ influx by fluorescence measurements	Dose-dependent induction of Ca^2+^ influx across the plasma membrane Inhibition of melittin-induced Ca^2+^ influx by OBAA	Catisti et al. (2000)
	R9 strain	Promastigotes	Cell viability analysis using MTT assay	High killing activity against promastigotes (LD_50_: 0.3 μM)	Díaz-Achirica et al. (1998)
	Not mentioned	Autoclaved promastigotes	Measurements of sLA-induced cytokines in the collected whole blood samples from mice receiving melittin-adsorbed autoclaved *L. donovani*	Significant reductions in the mean levels of IL-10 (*p* = 0.00001), IFN-γ (*p* = 0.00008) and TNF-α (*p* = 0.000001) in comparison to the control (non-treated) group	Eltahir Saeed and Khalil (2017)
*Leishmania infantum*	MHOM/BR/1972/LD strain	Amastigotes and promastigotes	Cell viability analysis using MTT assay, quantification of cytokines, and determination of NO and H_2_O_2_ production	Direct inhibition of both amastigotes and promastigotes Indirect inhibition of intracellular amastigotes by immunomodulatory effects on macrophages (increasing IL-12 production and decreasing the levels of IL-10, TNF- α, NO, and H_2_O_2_)	Pereira et al. (2016)
*Leishmania major*	Not mentioned	Promastigotes	Cell viability analysis using microplate Alamar blue assay	Induction of death in 50% of promastigotes at 74.01 ± 1.27 μg/mL	Pérez-Cordero et al. (2011)
*Leishmania panamensis*	Not mentioned	Promastigotes	Cell viability analysis using microplate Alamar blue assay	Ineffectiveness of melittin in killing 50% of promastigotes at > 100 μg/mL	Pérez-Cordero et al. (2011)
*Plasmodium berghei*	ANKA strain	Ookinetes and gametocytes	Analysis of the effects of melittin on ookinetes (in vitro) and sporogonic stages (*Anopheles stephensi*) of the parasite	Complete obliteration of ookinetes after 30 min Significant reductions in both infection prevalence (*p* = 0.019) and infection intensity (*p* < 0.001) compared to those in control mosquitoes	Carter et al. (2013)
*Plasmodium falciparum*	NF54 strain	Gametocytes	Analysis of the effects of melittin and multi-melittin arrays on sporogonic stages of *Anopheles coluzzii*	Significant reductions of infection intensity (*p* < 0.001) in mosquitoes fed on cultured *P. falciparum* spiked with melittin (native or modified peptide) compared to those in control mosquitoes	Habtewold et al. (2019)
	NF54 strain	Gametocytes	Analysis of the effects of melittin on sporogonic stages of *Anopheles gambiae*	Significant decrements in both infection prevalence (*p* < 0.001) and infection intensity (*p* = 0.019) compared to those in control mosquitoes	Carter et al. (2013)
*Toxoplasma gondii*	RHβ strain	Extracellular tachyzoites	β-galactosidase release assay for the assessment of lytic activity	Induction of cytosolic β-galactosidase release and cell lysis	Seeber, (2000)
*Trypanosoma brucei brucei*	M110 clone	Bloodstream forms	Evaluation of Ca^2+^ influx by fluorescence measurements	Dose-dependent induction of Ca^2+^ influx across the plasma membrane	Ruben et al. (1996)
	AnTat1.1E clone	Procyclic forms	Evaluation of Ca^2+^ movement between organelles by luminescence measurements	Transient retention of Ca^2+^ in mitochondria Contribution of acidic compartments to Ca^2+^ homeostasis during the signaling process	Xiong et al. (1997)
	ILTar 1 procyclics	Trypomastigotes	Evaluation of Ca^2+^ influx by fluorescence measurements	Dose-dependent induction of Ca^2+^ influx across the plasma membrane Inhibition of melittin effects on Ca^2+^ influx by OBAA, a PLA_2_ inhibitor Induction of Ca^2+^ release from intracellular stores in the absence of CaCl_2_ (and in the presence of 1 mM EGTA)	Catisti et al. (2000)
*Trypanosoma cruzi*	Y strain	Amastigotes	Evaluation of Ca^2+^ influx by fluorescence measurements	Induction of Ca^2+^ influx Inhibition of melittin effects on Ca^2+^ influx by OBAA	Catisti et al. (2000)
	macrophagotropic Tehuantepec strain	Trypomastigotes	Light, fluorescence, and electron microscopies, evaluation of trypanocidal activity, and measurement of β-galactosidase release (before and after parasitic infection)	Inhibition of the parasite motility Disruption of plasma membrane Reduction of the parasite infectivity No reduction in the growth of intracellular parasites	Jacobs et al. (2003)
	M/HOM/AR/74/CA-I CL72	Trypomastigotes	Determination of lethal concentration, evaluation of *T. cruzi* killing by dual peptide treatment, and recovery of AMP-treated cells after transfer to non-AMP containing media	High killing activity against *Trypanosoma cruzi* Synergistic and additive anti-parasitic effects of melittin in combination with certain AMPs Inability of the parasite to recover after treatment with 10 μM of melittin	Fieck et al. (2010)
	CL Brener clone	Epimastigotes and trypomastigotes	Evaluation of the parasite viability, flow cytometry analysis, and TEM	Dose-dependent decrease in the number of *T. cruzi* cells Permeabilization of protozoan cell membrane (High percentages of PI-labeled epimastigotes and trypomastigotes) Induction of autophagy (epimastigotes) and apoptosis (trypomastigotes)	Adade et al. (2012)
	CL Brener clone	Amastigotes, epimastigotes, and trypomastigotes	Evaluation of the parasite viability, treatment during the *T. cruzi* intracellular cycle, PI uptake assay, evaluation of mitochondrial membrane potential, TUNEL assay, SEM, TEM, and fluorometric analysis of MDC	Induction of growth inhibition or killing of developmental forms of the parasite (< 2.5 μg/mL) Induction of structural changes (plasma membrane blebbing, mitochondrial swelling, and nuclear alterations) Induction of alterations in *ΔΨm* Disorganization of the kinetoplast DNA filaments Induction of alterations in flagellar structure Permeabilization of cell membrane Induction of apoptosis and autophagy	Adade et al. (2013)

*ΔΨm* Mitochondrial membrane potential, *AMP* Anti-microbial peptide, *EGTA* Ethylene glycol-bis(β-aminoethyl ether)-*N*,*N*,*N*′,*N*′-tetraacetic acid, *H*_*2*_*O*_*2*_ Hydrogen peroxide, *IFN-γ* Interferon-γ, *IL-10* Interleukin-10, *IL-12* Interleukin-12, *LD*_*50*_ Half lethal dose, *MDC* Monodansylcadaverine, *MTT* 3-(4,5-dimethylthiazol-2-yl)-2,5-diphenyltetrazolium bromide, *NO* Nitric oxide, *OBAA* 3-(4-octadecyl)-benzoylacrylic acid; *PI* Propidium iodide, *PLA*_*2*_ Phospholipase A_2_, *SEM* Scanning electron microscopy; sLA: Soluble *Leishmania donovani* antigen, *TEM* Transmission electron microscopy, *TNF-α* Tumor necrosis factor-α, *TUNEL* Terminal deoxynucleotidyl transferase (TDT)-mediated dUTP-biotin nick end-labeling

Cytotoxicity assays provide a crucial means for safety assessment and screening in drug development (Fumarola et al. 2004). Various host cells have hitherto been subjected to different melittin concentrations. For instance, a 24-h incubation of immature human dendritic cells (10^5^ cells/mL) with melittin has unveiled a median lethal concentration (LC_50_) of 43.42 ± 0.86 μg/mL. A median hemolytic concentration (HC_50_) of 16.28 ± 0.17 μg/mL against human red blood cells (2% suspension) was also reported for the peptide (Pérez-Cordero et al. 2011). As regards mouse peritoneal macrophages (MPMs), an IC_50_ value of 5.73 μg/mL was obtained for melittin after 48 h. Despite complete eradication of *L. infantum* amastigotes, 2.5 μg/mL of melittin induced some morphological changes in MPMs (Pereira et al. 2016). Keep in mind that selective toxicity is a crucial feature of anti-microbial agents. It refers to a substance that is only toxic to a specified microorganism while inflicting minimal or no harm on the host cells (Bacalum and Radu 2015). Indeed, this can be assessed using selectivity index (SI), which is indicative of the therapeutic window of an anti-microbial agent. The higher the SI, the greater the difference between adverse effects and favorable anti-infective properties (Memariani et al. 2018). Comparing the toxic effects of melittin on MPMs with *L. infantum* amastigotes disclosed a SI value of 4 (Pereira et al. 2016). In other words, the cytotoxic activity of melittin against *L. infantum* is four times as high as that of the mammalian cells. Contrary to expectations, the SI value of ≤ 1 was shown for *L. panamensis* amastigotes and immature human dendritic cells, which was not satisfactory (Pérez-Cordero et al. 2011).

Macrophages are considered as pivotal host cells for *Leishmania* proliferation and elimination. *Leishmania* survival and persistence within the macrophages are known to be dependent upon several factors such as the species of the pathogen, type, and magnitude of the host immune responses (Tomiotto-Pellissier et al. 2018; Van Assche et al. 2011). Hence, proper activation of macrophages is an indispensable requirement for intracellular obliteration of *Leishmania*. Along with direct inhibitory effects, melittin seems to attenuate *Leishmania* infectivity through modulation of immune responses. Melittin influences the levels of anti-inflammatory [interleukin-10 (IL-10) and transforming growth factor- β (TGF-β)] and pro-inflammatory cytokines [interleukin-12 (IL-12) and tumor necrosis factor-α (TNF-α)] in vitro (Pereira et al. 2016). Noticeably, non-toxic doses of melittin was shown to augment IL-12 production in macrophages infected with *L. infantum*. IL-12 is believed to be necessary for the development of protective T-helper 1 (Th1)-predominant immunity as well as controlling the *Leishmania* proliferation (Okwor and Uzonna 2016; von Stebut et al. 1998). By virtue of the fact that IL-12 has the potential to act as an adjuvant in *Leishmania* vaccines (Scott and Trinchieri, 1997; Mutiso et al. 2010), melittin could be used in vaccine formulations to boost immune responses against leishmaniasis.

According to the work of Pereira et al. (2016), exposure of infected macrophages to melittin (2.5 μg/mL) for 24, 48, and 72 h resulted in a significant reduction in TNF-α levels compared to untreated infected macrophages (*p* < 0.05). Although TNF-α contributes to *Leishmania* clearance by enhancing macrophage activity and nitric oxide (NO) synthesis (Mirzaei et al. 2020), Pereira et al. (2016), however, were of the opinion that melittin-mediated downregulation of TNF-α might be beneficial in mitigating untoward effects of TNF-α excess. Beside this, a notable drop in IL-10 levels was evident in the melittin-treated infected macrophages (Pereira et al. 2016). Considering the role of IL-10 in macrophage deactivation and parasite persistence (Kane and Mosser, 2001; Mirzaei et al. 2020), reduction of IL-10 levels by melittin might thwart the disease progression. By contrast, when the infected macrophages were challenged with different doses of melittin, no variation was observed in TGF-β levels. In comparison to the untreated infected macrophages, incubation of the infected macrophages with melittin caused significant diminution in the levels of NO and hydrogen peroxide (H_2_O_2_) (*p* < 0.05). These results suggest that the eradication of intracellular amastigotes by melittin might occur in a H_2_O_2_- and NO-independent mechanism (Pereira et al. 2016).

In a study conducted by Eltahir Saeed and Khalil (2017), Swiss CD1 mice were injected with three intradermal doses of melittin-adsorbed autoclaved *L. donovani* (ALD). The collected whole blood samples from these mice were then stimulated with soluble *L. donovani* antigen (sLA), after which the mean levels of some cytokines in cell supernatants were measured. Interestingly, the mean levels of sLA-induced IL-10, Interferon-γ (IFN-γ), and TNF-α were found to be substantially greater in the blood samples of aforementioned mice than those of the control (non-treated) group (Eltahir Saeed and Khalil 2017). The observation that melittin reduces IL-10 levels is consistent with the earlier findings by Pereira et al. (2016). Based on these results, the conclusion was drawn that melittin could modulate both Th1 and Th2 immune responses in Swiss CD1 mice (Eltahir Saeed and Khalil 2017). Taken together, melittin-adsorbed autoclaved *Leishmania* has the potentiality for developing anti-leishmania vaccine.

### *Trypanosoma brucei*

Similar to *Leishmania*, *Trypanosoma* is a genus of kinetoplastids, a group of unicellular flagellated eukaryotes related to the euglenids (Stuart et al. 2008). Human African trypanosomiasis (HAT) or sleeping sickness is a deadly insect-borne disease that flourishes in impoverished, rural parts of sub-Saharan Africa, where it is transmitted by the bite of tsetse fly (Bukachi et al. 2018). Two sub-species of *Trypanosoma brucei* are responsible for the disease in humans. *Trypanosoma brucei gambiense* gives rise to slow-onset chronic illness in western and central Africa, whereas *Trypanosoma brucei rhodesiense* is associated with a more acute form of HAT in southern and eastern Africa. The third subspecies *Trypanosoma brucei brucei* is a causative agent of animal trypanosomiasis, and does not infect humans (Malvy and Chappuis 2011). Trypanosomes are shrouded in a variant surface glycoprotein coat, helping them to escape the host immune reactions. Owing to the sophisticated and evasive nature of the pathogen, vaccination against *T. brucei* has been futile (Black and Mansfield 2016). Thus far, only four medications are registered for the treatment of early- and late-stage HAT: pentamidine, suramin, melarsoprol, and eflornithine. Although nifurtimox is not approved for chemotherapy, it has been used in combination with eflornithine for the second stage of HAT due to *T.b. gambiense* (Malvy and Chappuis 2011).

During the late 1990s, a number of studies provided compelling evidence that melittin rises [Ca^2+^]i in *T. b. brucei* (Ruben et al. 1996; Xiong et al. 1997; Ridgley et al. 1999). The initial indication of melittin-induced Ca^2+^ influx across the parasite plasma membrane came from two different experiments which were undertaken by Ruben et al. (1996). In the first experiment, melittin had no impact on [Ca^2+^]i in a buffered salt solution comprising 3 mM ethylene glycol-bis(β-aminoethyl ether)-*N*,*N*,*N*′,*N*′-tetraacetic acid (EGTA; for chelating the extracellular Ca^2+^). The peptide, however, caused an elevation of [Ca^2+^]i in the same solution containing 2 mM Ca^2+^. Further evidence in support of this observation was gained through Mn^2+^ quench experiments on *T. b. brucei* cells loaded with calcium-sensitive dye Fura-2. Analogous findings relative to the melittin-induced Ca^2+^ influx in *T. b. brucei* cells were described by Catisti et al. (2000). Plasma membrane-located Ca^2+^ channels, but not permeabilized plasma membrane, appeared to be contributing to the aforesaid Ca^2+^ influx (Ruben et al. 1996). The enthused readers ought to consult the original paper for further details (Ruben et al. 1996).

A subsequent study by Xiong et al. (1997) extended the above-mentioned findings to indicate that most of the Ca^2+^, which were entered the cell across the plasma membrane or were liberated from the acidocalcisome, transiently accumulated into mitochondria during the signaling process induced by melittin. In another investigation, melittin (200 nM) was shown to impair the Ca^2+^ transport properties of the mitochondria (Ridgley et al. 1999). In response to melittin, the acidocalcisome thought to be involved in maintaining Ca^2+^ homeostasis (Xiong et al. 1997). These findings were further confirmed by a study in which exposure of *T. b. brucei* procyclic trypomastigotes to melittin in Ca^2+^-free medium led to an appreciable increase in [Ca^2+^]i (Catisti et al. 2000). This highlights the probable role of intracellular Ca^2+^ stores such as the acidocalcisomes in Ca^2+^ mobilization. Considering the destabilization effects of melittin on [Ca^2+^]i in *T. brucei* cells, it will be of interest to utilize melittin and other anti-trypanosomatid drugs acting on Ca^2+^ homeostasis concurrently to comprehend if synergism occurs.

Hydrolysis of phosphatidylinositol-4,5-bisphosphate by phospholipase C yields inositol-1,4,5-trisphosphate (IP_3_) and diacylglycerol, both of which act as second messengers in eukaryotic signal transduction pathways (Cestari 2020; de Paulo Martins et al. 2010). The former prompts Ca^2+^ release from intracellular stores, while the latter activates protein kinase C (Catisti et al. 2000). Ruben et al. (1996) quantitated IP_3_ in control and melittin-treated *T. b. brucei* cells individually. They found no substantial changes in the resting levels of IP_3_ after 30-s or 2-min exposure of *T. b. brucei* cells to melittin. Their conclusion was that the trypanosomal calcium influx induced by melittin appeared to be independent of IP_3_ involvement. Furthermore, the effect of melittin on [Ca^2+^]i could be mediated by a phospholipase A_2_ (PLA_2_) activation (Catisti et al. 2000). In support of this, it was shown that melittin-induced Ca^2+^ influx in procyclic trypomastigotes of *T. b. brucei* was inhibited using 5 μM of 3-(4-octadecyl)-benzoylacrylic acid (OBAA), a PLA_2_ inhibitor (Catisti et al. 2000).

It has been demonstrated that mammalian cells are less vulnerable to melittin treatment in comparison with *T. b. brucei*. In this respect, the dose at which the parasite gave a response was 5–20 times lower than that of mammalian cells (Ruben et al. 1996). The reason for this observation is as yet unclear, but it might be attributable to some differences between Ca^2+^ channels, contributing to intracellular Ca^2+^ homeostasis, in mammalian and *T. b. brucei* cells. Indeed, Ca^2+^ plays a pivotal part in regulation of multiple biological processes in trypanosomes such as invasion, cellular differentiation, and flagellar movements, to cite only a few (Benaim et al. 2020; Smirlis et al. 2010). In light of the fact that melittin induces [Ca^2+^]i disturbance in *T. brucei* cells, it would be desirable to assess whether or not the peptide affects above-mentioned processes.

### *Trypanosoma cruzi*

*Trypanosoma cruzi* is the causative agent of Chagas disease, which is largely confined to endemic regions of Latin American countries (Harrison et al. 2020). Contamination of the bite site or intact mucous membranes by infected triatomine bug feces is the primary route of transmission (Bern 2015). Other less common routes of transmission include blood transfusions, organ transplantation, and transplacental transmission. If the disease progresses to the chronic phase, serious complications such as congestive heart failure, esophageal dilatation, and enlargement of colon may occur (Bern 2015). Benznidazole and nifurtimox are the only medications with proven efficacy against Chagas disease; however, both drugs exhibit significant adverse effects and low effectiveness in adults with chronic infections (Villalta and Rachakonda 2019).

There are multiple lines of evidence affirming the anti-parasitic effects of melittin upon T. *cruzi* (Table 1). For instance, IC_50_ of *T. cruzi* epimastigotes was 2.44 ± 0.23 μg/mL after a single day of incubation with melittin, while this value was much lower (0.22 ± 0.09 μg/mL) for intracellular amastigote (Adade et al. 2013). Using light microscopy, melittin (2.5 μM) was shown to cease the motility of at least 50% of *T. cruzi* after 30 min of incubation (Jacobs et al. 2003). This event might result from alterations in flagellar structure and/or direct lethal activity of melittin. In connection with the former, melittin was shown to induce multiple morphological abnormalities in flagella such as swelling in some region of the flagellum, formation of cracks, and blebbing of flagellar membrane (Adade et al. 2013). Moreover, some authors have pointed out that melittin possesses lethal activity against *T. cruzi* in vitro (Fieck et al. 2010; Adade et al. 2012, 2013). For example, Fieck et al. (2010) demonstrated that melittin had a lethal concentration (LD_100_) of 30 μM towards *T. cruzi* after 96 h.

The most frequent cell lines that have been exploited as host cells for *T. cruzi* studies are Vero (African green monkey renal epithelial cells), LLC-MK2 (Rhesus monkey kidney epithelial cells), peripheral blood mononuclear cells (PBMCs), and human placenta derivatives (Duran-Rehbein et al. 2014). Neither un-infected LLC-MK2 cells nor un-infected mouse peritoneal macrophages were found to be cytotoxically affected by 1 μg/mL of melittin after 24 h. In LLC-MK2 cells, however, increasing melittin concentration to 5 μg/mL led to 49% cell death (Adade et al. 2013). The selectivity indices of melittin, measured by the ratio between the peptide toxicity to LLC-MK2 and parasite cells, were calculated to be 2.05, 35.7, 22.7 for epimastigotes, trypomastigotes, and intracellular amastigotes, respectively (Adade et al. 2013). From this finding, epimastigotes appear to be more efficiently inhibited by melittin in comparison with trypomastigotes and intracellular amastigotes. Nevertheless, all three developmental forms of *T. cruzi* can be inhibited/killed by melittin more selectively than the host cells. The observed selective toxicity might be ascribed to the inherent differences between biomembrane lipid compositions of the host cells and the parasite, in particular different quantities of total phospholipids and sterols (Souza et al. 2016). In one study, pre-incubation of *T. cruzi* with as low as 1 μM melittin considerably curtailed the ability of parasite to infect human glioblastoma cell line 86HG39 (Jacobs et al. 2003). In spite of this, melittin failed to abolish intracellular growth of *T. cruzi* in infected 86HG39 cells after 24 h (Jacobs et al. 2003).

It appears as though programmed cell death (PCD) plays a role in the control of proliferation and differentiation in trypanosomatids (Piacenza et al. 2007; Lee et al. 2002). PCD has been characterized on the basis of morphological criteria and environmental conditions. This process can be categorized into three distinct types, namely apoptosis (type I PCD), autophagy (type II PCD), and programmed necrosis (type III PCD) (Kroemer et al. 2009; Adade et al. 2013). In trypanosomatids, a wide range of stress conditions such as anti-protozoal drugs and nutrient depletion were proved to be involved in autophagy (Menna-Barreto 2019). In 2012, Adade et al. (2012) were the first to declare that melittin instigated dissimilar PCD pathways in epimastigotes and trypomastigotes at concentrations that were non-toxic for peritoneal macrophages. In this sense, autophagy and apoptosis appeared to be the frequent causes of cell death in epimastigotes and trypomastigotes, respectively. Shortly thereafter, these investigators (Adade et al. 2013) succeeded in affording more details upon the lethal effects of melittin towards all *T. cruzi* developmental forms (Table 1). Some ultrastructure alternations in the melittin-treated epimastigotes involve mitochondrial swelling and the appearance of endoplasmic reticulum profiles around various organelles (resembling autophagy), which were analogous to those observed in melittin-treated amastigotes. In the case of trypomastigotes, melittin was shown to induce mitochondrial swelling, kinetoplast DNA (kDNA) disorganization, and nuclear apoptotic changes (Adade et al. 2013).

Terminal deoxynucleotidyl transferase (TDT)–mediated dUTP-biotin nick end-labeling (TUNEL) assay is a technique for detection of DNA fragmentation in apoptotic cells (Kyrylkova et al. 2012). For staining of autophagic vacuoles, an autofluorescent lysosomotropic substance named monodansylcadaverine (MDC) is commonly used (Biederbick et al. 1995). Using TUNEL assay and MDC labeling of *T. cruzi*, Adede et al. (2013) were able to further substantiated differences in PCD pathways of melittin-treated epimastigotes and trypomastigotes. Contrary to melittin-treated epimastigotes, DNA fragmentation was more pronounced in melittin-treated trypomastigotes, which is reminiscent of an apoptosis-like death. However, exposure to melittin caused a significantly higher MDC fluorescence intensity in epimastigotes compared to trypomastigotes (*p* ≤ 0.05), indicating a predominance of autophagic-like cell death in epimastigotes (Adade et al. 2013).

In a study conducted by Jacobs et al. (2003), treatment of β-galactosidase-expressing trypomastigotes with 5 μM melittin evoked β-galactosidase release into the supernatant, particularly after 15 min. The same authors further noted that melittin brought about a total disruption of the parasite plasma membrane. In accordance with this observation, a 1-day melittin treatment of epimastigotes (IC_50_) or trypomastigotes (LD_50_) resulted in a marked increase in the number of propidium iodide (PI)-labled cells (Adade et al. 2013). PI is an intercalating DNA-binding dye, and is indeed capable of entering and staining cells having compromised membrane integrity (Memariani et al. 2020b). Apart from disrupting cell membrane integrity, melittin may reduce mitochondrial membrane potential (*ΔΨm*) (Adade et al. 2013).

Melittin can act synergistically or additively with certain AMPs to eradicate *T. cruzi* cells in vitro. When used in paired treatments, melittin in combination with magainin II exhibited synergistic interactions. Other AMPs such as apidaecin and cecropin A had additive effects. *T. cruzi* cells subjected to these treatments were unable to recover after transfer to AMP-free media for 96 h (Fieck et al. 2010), attesting to the potential utility of melittin-AMP mixtures as efficacious trypanocidal agents. Curiously, LD_100_ value for melittin against *T. cruzi* was 2.6 times as low as the minimal bactericidal concentration for *Rhodococcus rhodnii*, an obligate symbiotic bacterium in the midgut of Chagas disease vector *Rhodnius prolixus* (Fieck et al. 2010). Based on these findings, melittin, either alone or combined with other effector molecules, would seem to be a propitious candidate for future paratransgenic systems to control transmission of Chagas disease.

### *Plasmodium* spp.

The phylum Apicomplexa constitutes an extremely large and diverse group of obligatory parasites, which has developed extraordinary adaptations for invading and surviving within their hosts (Suarez et al. 2019). *Plasmodium*, *Toxoplasma*, *Cryptosporidium*, *Eimeria*, *Neospora*, *Theileria*, and *Babesia* are the most pervasive apicomplexans in mammalian hosts (Seeber and Steinfelder, 2016). Four species of *Plasmodium*, the causative agent of malaria, have long been considered true parasites of humans: *P. falciparum*, *P. malariae*, *P. ovale*, and *P. vivax*. Malaria is a life-threatening mosquito-borne disease that inflicted a tremendous burden on many tropical countries (Talapko et al. 2019). In 2018, there were an estimated 228 million cases of malaria occurred globally, of whom around 405,000 succumbed to death (WHO 2019). Various *Plasmodium* species exhibit different clinical presentations, progression, and anti-malarial resistance patterns. The deadliest species is *P. falciparum*, accountable for the vast majority of the mortality and morbidity associated with malaria infection (Phillips et al. 2017). Quinine derivatives and artemisinin compounds are crucial anti-malarial medications. Disappointingly, the emergence and dissemination of resistance against these drugs have thwarted efforts to control human malaria. On top of that, development of a malaria vaccine is technically very challenging because the pathogen has evolved highly effective immune-evasion strategies (Ashley et al. 2018).

*Plasmodium berghei*, a rodent pathogen, has been frequently used as a model microorganism for human malaria studies (Goodman et al. 2013). In a study carried out by Carter et al. (2013), a 30-min incubation of *P. berghei* ookinetes with 50 μM of melittin culminated in a complete loss of parasite viability. However, melittin (25 μM) showed profound toxicity to an *Anopheles gambiae* cell line (Sua 4.0) after 3 h of incubation. Notwithstanding this in vitro cytotoxicity, feeding blood containing 50 μM of melittin to mosquitos had no significant deleterious effects on either longevity or fecundity over a 10-day period (Carter et al. 2013). One possible explanation for these discrepant findings is that there exist some physiological differences between cultured cells and midgut epithelial cells of mosquitos. For instance, the peritrophic matrix might protect the latter from mechanical and chemical damages (Parish et al. 2011; Lehane 1997).

A promising approach to control *Plasmodium* transmission is the production of genetically modified vectors (transgenic organisms) that are incapable of malaria transmission (Ogaugwu and Durvasula, 2017). This might be achieved through interference with the malaria mosquito's vectorial capacity to support* Plasmodium* development (Wang et al. 2017). It has also been suggested that the midgut lumen of anopheline vector can serve as a prime target for relevant interventions since this hostile environment represents a severe bottleneck to parasite transmission (Paton et al. 2019; Wang and Jacobs-Lorena, 2013). A perfect transmission-blocking molecule selected to be expressed in midgut of genetically modified vectors needs to be highly efficacious, soluble, rapid-acting, and resistant to proteolytic degradation while having no negative impact upon both lifespan and reproductive abilities of the mosquito (Carter and Hurd, 2010). Furthermore, the ability of an effector molecule to interrupt parasite transmission can be quantified by infection prevalence and infection intensity (Wu et al. 2015). The former denotes the proportion of mosquitoes harboring at least one oocyst, while the latter implies the number of oocysts per mosquito (Habtewold et al. 2019). In this regard, one study assessed the in vivo effects of several anti-microbial peptides (AMPs) against sporogonic stages of *P. berghei* when *Anopheles stephensi* were fed on gametocyte-containing blood supplemented with 50 μM of each AMP separately (Carter et al. 2013). Among tested AMPs, melittin was the only effective peptide that significantly diminished parasite prevalence by an average of 10% and intensity of infection by 68%. Almost similar trends were also evident when melittin was tested against the sporogonic stages of *P. falciparum* (Carter et al. 2013).

Particularly impressive was the recent work in which a streamlined and robust standard membrane feeding assay (SMFA) protocol employing coordinated culturing of *Anopheles coluzzii* and *P. falciparum* gametocyte was devised by Habtewold et al. (2019). This SMFA protocol consistently yielded high oocyst intensities and prevalence, hence permitting precise appraisal of the effectiveness of transmission-blocking interventions. Using the above-mentioned protocol, the same authors were able to assess the transmission-blocking potential of six selected AMPs, which had previously been shown to exert anti-protozoan activities towards blood-stage *P. falciparum* (Habtewold et al. 2019). When added to gametocytaemic blood, melittin (50 μM) exhibited a significant transmission-blocking activity (*p* < 0.001) in comparison to PBS-treated mosquitoes (control). The authors also evaluated the expression of multi-melittin arrays separated through 2A autocleavage peptides or a furin cleavage site in transgenic mosquitoes. It is worth mentioning that the processed peptides following the 2A or furin cleavage would be expected to have further amino acid residues (Wang et al. 2015; Liu et al. 2017), which might modify the structural and functional properties of the expressed peptides. The experimental data revealed that the presence of additional amino acids in expressed melittin had no substantial negative impact upon its transmission-blocking activity, and indeed could potentiate it, as is the case of melittin with sequence “EENPG” at its C-terminus (Habtewold et al. 2019). Overall, transgenic mosquitoes expressing AMPs may offer opportunities for controlling malaria.

### *Toxoplasma gondii*

*Toxoplasma gondii* is an obligate intracellular parasite that infects many warm-blooded animals including birds, mammals, and humans. Like other Apicomplexan parasites, *T. gondii* uses sophisticated, ingenious strategies to invade their host cells (Francia et al. 2016; Reiling and Dixon, 2019). Felines are known to be the only definitive hosts of the pathogen. Infection usually occurs by consuming undercooked contaminated meat harboring tissue cysts, contacting with infected cat feces, mother-to-child transmission during pregnancy, and through blood transfusion or organ transplantation (Robert-Gangneux and Dardé, 2012). Almost one third of the world's population who are infected with *T.* *gondii* remain asymptomatic. Nonetheless, a small percentage of these patients, in particular immunodeficient individuals, manifest severe disease. In humans, *T. gondii* may form tissue cysts in muscles, myocardium, brain, and eyes (Flegr et al. 2014). Due to the parasite’s fondness for brain and retinal tissue, infections may result in chronic complications such as blindness or neurological abnormalities (Flegr et al. 2014). Although the disease is usually self-limiting, clinically severe or persistent toxoplasmosis can be treated with a combination of drugs such as pyrimethamine and sulfadiazine, which inhibits parasite folate metabolism (Rajapakse et al. 2013). The need for long-term therapy and the risk of relapsing disease may be attributed to the lack of effectiveness of these medications towards *T. gondii* cysts (Alday and Doggett, 2017).

Anti-protozoan effects of melittin against *Toxoplasma gondii* have been seldom explored. In one study, for instance, Seeber (2000) employed a method in which the membrane lytic effect of melittin on *lacZ* transgenic strain of *T. gondii* (RHβ-1) could be determined by measuring the activity of liberated cytosolic β-galactosidase into the culture supernatant. The author showed that there was a correlation between β-galactosidase activity and the number of extracellular *T. gondii* tachyzoites. Incubation of purified extracellular tachyzoites with 1.75 μM melittin for 1 h at 37 °C led to a noticeable increase in β-galactosidase release, which was approximately similar to that of 0.25% Triton X-100 (Seeber 2000). This finding implies that melittin directly eradicates extracellular *T. gondii* tachyzoites through disruption of plasma membrane integrity. Though these results appear promising, further research is required to characterize the anti-protozoan effects of melittin on different developmental forms (i.e. bradyzoites and sporozoites) of the parasite.

## Future prospects

**Fig. 1:**
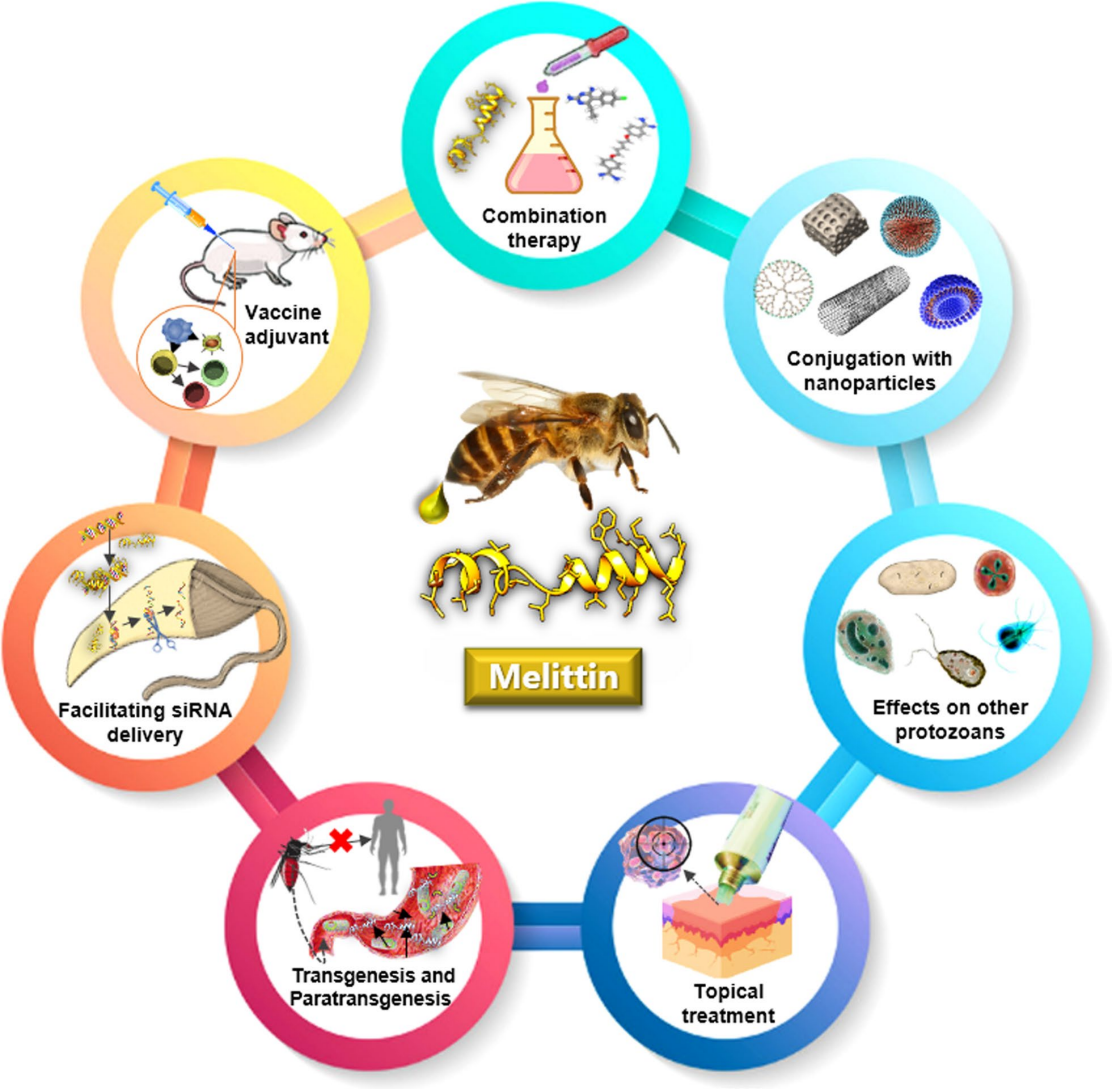
Potential biomedical applications of melittin for future studies on protozoan diseases

Discovery of venom-derived AMPs has given a renewed impetus to anti-parasitic drug development. Despite potent anti-protozoan effects of melittin, the efficacy of the peptide in animal models should be further scrutinized in future investigations. Pre-clinical studies will confront several challenges such as cytotoxicity, in vivo stability, and routes of administration. We envisage that conjugation of melittin with nanoparticles holds great promise in different biomedical applications. Not only does this approach improve the target-specific delivery of melittin with less cytotoxicity, but it also enhances proteolytic stability of nanoparticle-melittin constructs. Given that melittin and its derivatives can act as cell‐penetrating peptides (Hou et al. 2013), they may facilitate small interfering RNA (siRNA) transfection for the purpose of suppressing expression of virulence genes in protozoan pathogens (Fig. 1).

Synergistic interactions between melittin and frequently used antibiotics have previously demonstrated evidence of efficacy against bacterial pathogens (Memariani et al. 2019). The ability to combine melittin with established anti-protozoan drugs bodes well for the future. Melittin even at sub-toxic concentrations may boost therapeutic potential of the current medications. Deployment of DNA microarrays and real-time polymerase chain reaction (PCR) assays to evaluate expression levels of many genes in pathogens after melittin treatment and drug-target identification will surely expand our knowledge with reagard to possible cellular responses induced by the peptide challenge. Furthermore, it deserves emphasis that a melittin-based topical cream or ointment could be propounded as a promising treatment for a wide spectrum of dermal infections, from ringworm (dermatophytosis) and wart to cutaneous leishmaniasis (Fig. 1).

Different bee venom components possess immunostimulatory or immunosuppressive properties, depending on dose, time, and the route of administration. Administration of melittin in combination with conventional vaccines can enhance both cell-mediated and humoral immune responses (Fig. 1). Melittin has been proposed as an adjuvant for leishmaniasis (Eltahir Saeed and Khalil, 2017), hepatitis B (Dezfuli et al. 2014), and tetanus-diphtheria vaccines (Bramwell et al. 2003). Nevertheless, further studies are needed to confirm effectiveness of such vaccines in vivo.

The possibility of controlling arthropod-borne diseases through vector transgenesis has recently garnered popular support and is being actively pursued by a number of research laboratories across the globe (Thomas 2018). These genetically modified invertebrates are capable of hampering parasite development by tissue-specific expression of effector molecules that impair pathogen adhesion to the midgut of vectors or activate biochemical pathways detrimental to survivability of pathogens (Coutinho-Abreu et al. 2010). In the paratransgenic strategy, however, genetically altered symbionts are re-introduced back to the vector where expression of the effectors interferes with pathogen transmission (Hurwitz et al. 2012). Various effectors, including AMPs and highly specific single chain antibodies, have been previously explored for controlling vector-borne diseases (Hurwitz et al. 2012; Giovati et al. 2018). Melittin, alone or in combination with other AMPs, has been nominated for both transgenic and paratransgenic strategies (Fieck et al. 2010; Ogaugwu and Durvasula, 2017).

## Conclusion

The growing problem of drug resistance among protozoan pathogens together with the dearth of new anti-parasitic medications poses a major public health challenge. Over the past years, melittin has gained a great deal of attention for its potent anti-protozoan properties. Accumulating evidence suggests that many protozoan parasites such as *Leishmania*, *Plasmodium*, *Trypanosoma*, and *Toxoplasma* are susceptible to melittin at micromolar concentrations. Melittin obliterates protozoan pathogens by several mechanisms of action including, but not limited to, disruption of cell membrane, destabilization of calcium homeostasis, reduction in mitochondrial membrane potential, and induction of different PCD pathways. Interestingly, production of genetically engineered symbiotic bacteria or transgenic invertebrates expressing melittin appears to be a promising strategy for inhibiting the transmission of vector-borne protozoan diseases. Taken altogether, there is no doubt that melittin would herald a new horizon in the fight against microbial pathogens.

## Data Availability

Not applicable.

## Abbreviations

*ΔΨm*: Mitochondrial membrane potential
ALD: Autoclaved *Leishmania donovani*
AMPs: Anti-microbial peptides
[Ca^2^^+^]i: Intracellular concentration of free calcium
EC_50_: Median effective concentration
EGTA: Ethylene glycol-bis(β-aminoethyl ether)-*N*,*N*,*N*′,*N*′-tetraacetic acid
HAT: Human African trypanosomiasis
HC_50_: Median hemolytic concentration
H_2_O_2_: Hydrogen peroxide
IFN-γ: Interferon-γ
IL-10: Interleukin-10
IL-12: Interleukin-12
IP_3_: Inositol-1,4,5-trisphosphate
LD_50_: Half lethal dose
MDC: Monodansylcadaverine
MPMs: Mouse peritoneal macrophages
MTT: 3-(4,5-Dimethylthiazol-2-yl)-2,5-diphenyltetrazolium bromide
NO: Nitric oxide
OBAA: 3-(4-Octadecyl)-benzoylacrylic acid
PCD: Programmed cell death
PI: Propidium iodide
PLA_2_: Phospholipase A_2_
SI: Selectivity index
sLA: Soluble *Leishmania donovani* antigen
SMFA: Standard membrane feeding assay
TGF-β: Transforming growth factor-β
Th1: T-helper 1
TNF-α: Tumor necrosis factor-α
TUNEL: Terminal deoxynucleotidyl transferase (TDT)–mediated dUTP-biotin nick end-labeling
